# Clinico-pathologic spectrum of C3 glomerulopathy-an Indian experience

**DOI:** 10.1186/s13000-015-0233-0

**Published:** 2015-03-17

**Authors:** Ganesh Kumar Viswanathan, Ritambhra Nada, Ashwani Kumar, Raja Ramachandran, Charan Singh Rayat, Vivekanand Jha, Vinay Sakhuja, Kusum Joshi

**Affiliations:** Department of Histopathology, Post Graduate Institute of Medical Education and Research, Chandigarh, 160012 India; Department of Nephrology, Post Graduate Institute of Medical Education and Research, Chandigarh, 160012 India

**Keywords:** Alternate complement pathway, C3 glomerulopathy, Complement, Dense deposit disease, Nephrotic, Nephritic

## Abstract

**Background:**

C3 glomerulopathy (C3GP) is characterized by deposition of complement C3 with absence/traces of immunoglobulins in the glomeruli and categorized into dense deposit disease (DDD), C3 glomerulonephritis (C3GN), complement factor H related protein 5(CFHR5) nephropathy etc. Collaborative efforts of pathologists, complement biologists and nephrologists worldwide are expanding the histomorphological pattern and laboratory findings related to C3GP. Hence, we studied point prevalence and morphological spectrum of C3GP in Indian patients to correlate morphological patterns with standard therapies and outcome of the patients.

**Methods:**

Retrospective analysis of renal biopsies (2007-2012,n-4565), which on immunofluorescence (IF) had C3 dominant deposits with absence or trace amount of immunoglobulin was carried out. Histopathology and electronmicroscopy (EM) were reviewed; cases were re-classified as DDD and C3GN. Histomorphological patterns of both groups were compared and correlated with treatment. Clinical details and follow up of patients were retrieved from the department of nephrology.

**Results:**

There were 31 cases (0.7%) of C3GP sub-classified as DDD (n-13) and C3GN (n-14). It was difficult to sub-classify 4 cases since EM showed overlapping features. C3GN and DDD had distinct clinical characteristics and disease outcome, though pathological features were overlapping. Majority of C3GP patients were males and were in 2^nd^ to 4^th^ decade of life. Nephrotic syndrome in DDD and nephritic-nephrotic presentation in C3GN patients was more common.

Hypertension and oliguria were more often observed in C3GN than DDD. Membranoproliferative pattern (MPGN) was commonest pattern in DDD; other patterns seen were mesangial proliferative, mesangial expansive/nodular, exudative and crescentic. C3GN also had all the above patterns, the predominant ones being MPGN and mesangial proliferative. Limited follow-up revealed response to therapy only in C3GN (33%). Progression to ESRD was 33% in DDD and 10% cases in C3GN.

**Conclusion:**

C3GP comprise 0.7% of all renal biopsies. MPGN pattern was the commonest morphological pattern in DDD whereas MPGN and mesangial proliferative pattern were equally dominant patterns in C3GN. EM of 4 cases (13%) showed intermediate features. Evaluation of alternate complement pathway must be done in all cases to identify the point of dysregulated alternate complement pathway and to confirm the diagnosis in ambiguous cases.

**Virtual slides:**

The virtual slides for this article can be found here: http://www.diagnosticpathology.diagnomx.eu/vs/1730070964135632

## Background

C3 glomerulopathy (G3GP) is a term coined for glomerular diseases characterized by accumulation of complement C3 with absence or trace amount of immunoglobulin deposition in the glomeruli [1]. Further sub-characterization of C3GP into its subtypes i.e. DDD and C3GN require LM/EM. Genetic evaluation is required for CFHR5 nephropathy [2-4]. Sethi et al. conceptualised evolution of C3GP based on their proteomic analysis of deposits in the renal biopsies of MPGN type 1, DDD [5] and later C3GN [6-8]. By advanced mass spectrometry techniques on glomerular isolates, *Sethi et al.* [8], documented that deposits in DDD were composed of activated components of the alternative pathway and lacked immunoglobulins. They also demonstrated the presence of component of the classic pathway in immune-complex mediated MPGN type-1. *Sethi et al*. therefore, proposed a classification driven by these findings on immunofluorescence that had the correlation with pathogenetic pathway of the disease under evaluation, classifying MPGN as either immunoglobulin positive or negative [2]. During the same period another entity C3GN was categorized which was also had same proteomic profile as of DDD and concept of C3GP evolved [6,8-10].

Earlier, based upon the localization of deposits by electron microscopy, primary MPGN were classified into three types. These three were type-I (subendothelial immune-complex type deposits), type-II (intramembranous osmiophilic) and type-III (immune-complex type subendothelial and subepithelial deposits) [3,9]. Type-I and III were typically immunoglobulin mediated diseases caused by the deposition of immune complexes and related with activation of classical complement pathway. MPGN type-II (also known as DDD) was characterized by intramembranous and mesangial osmiophilic electron-dense deposits on electron microscopy. In DDD, deposition of complement products were seen in immunofluorescence with absence or trace amount of immunoglobulin [1,2].

Besides the immunohistology, abnormalities in alternate pathway function were identified as a cause of C3GP in complement-mediated MPGN. Several mutations in alternative pathway inhibitors were documented which were responsible for the aetiopathogenesis of these diseases. Several autoantibodies responsible for the activation or blockage of alternative pathway proteins were also documented which contributed significantly to this evolving concept [4,7,9].

Though, the concept of C3GP evolved from analysis of mesangiocapillary pattern (MCGN), now is known to have varied histomorphology including MPGN pattern, crescentic or diffuse proliferative glomerulonephritis [3,11,12].

It is important to diagnose and segregate patients of C3GP from other glomerular diseases with similar morphological patterns as targets for therapy are different. Immune complex-mediated disease needs targeting primary infection, relevant autoantibodies or clonal light chains according to the diagnosis. In C3GP, plasmapheresis for removal of autoantibodies and recently use of Ecluzimab [13], targeted against C5 component are the only potential available targets. Variants of C3GP also important from renal transplantation perspective as rate of recurrence are variable with different subtypes of C3GP [13,14]. One variant of C3GP has geographic/ethnic aggregation [15,16] (Cyprus).

There is no literature on C3GP from India. In present pilot study, we evaluated all renal biopsies which had only C3 deposition to find histological pattern of C3GP. Our institute is a tertiary health care centre located in north India catering largely to north Indian patients. However, since it is fully equipped referral centre with all the facilities including EM, we also get referred cases of renal diseases from other states like Utter Pradesh, Bihar, West Bengal, Assam, etc. Our patient cohort might be representative of Indian patients in the context of this group of renal diseases.

## Methods

This study was carried out in the department of histopathology, Post Graduate Institute Of Medical Education And Research, (PGIMER) Chandigarh. Study period was of 5 years (second half of 2007 to the first half of 2012). It included both retrospective and prospective data. The clinical details retrieved from the records of the department of nephrology, PGIMER, Chandigarh. Details of kidney biopsies (LM, IF and EM reports) retrieved from the records of the department of histopathology, PGIMER, Chandigarh. Starting material for this study was selected by scrutinizing IF records and photomicrographs, cases with only C3 deposits in the absence of immunoglobulins for further analysis. Haematoxylin and eosin and periodic acid Schiff stained slides were reviewed by two nephropathologists to characterize the morphology, and electron microscopic photographs reviewed for different morphological patterns of C3GP.

### Cases with C3 deposits classified into following morphological patterns

- **MPGN** - presence of endocapillary proliferation with global duplicated basement membranes
- **Mesangial proliferation** - mesangial proliferation with normal glomerular membranes
- **Exudative/DPGN like** - in addition to mesangiocapillary pattern presence of numerous polymorphs and/or eosinophils in the glomeruli
- **Mesangial expansive** - includes nodular and non-nodular mesangial expansive pattern
- **Crescentic** - >50% of the total number of glomeruli show crescents.

Number of sclerosed glomeruli were recorded. The tubular atrophy and interstitial inflammation were recorded and graded on four step scale (Grade 0 – None, Grade 1 - <33%, Grade 2 - 33-66%, Grade 3 - >66%). Presence of interstitial foam cells, refractile thickening and vascular changes were recorded (Grade 0 – Absent, Grade 1 – present).

### Definition

#### Nephrotic syndrome

Proteinuria ≥ 3.5 g/day or ≥ 1.5 g/d, serum albumin < 2.5 g/dl, edema, and hyperlipidemia with or without hematuria (>5 RBCs/ HPF).

#### Nephroto-nephritic syndrome

Proteinuria ≥ 3.5 g/d or ≥ 1.5 g/d along with serum albumin < 2.5 g/dl, edema, hyperlipidemia with or without hematuria (>5 RBCs/ HPF), hypertension (BP > 140/90 mm Hg) and renal dysfunction (eGFR 15-60 ml/min).

#### Advanced renal failure

Dialysis dependent renal failure or eGFR < 15 ml/min.

#### Complete remission

Reduction of proteinuria to < 0.3 g/d and with creatinine clearance of > 60 ml/min/1.73m^2^ and serum albumin > 3.5gm/dl.

#### Partial remission

Reduction of proteinuria to 0.3–3.5 g/day, stable serum creatinine (change in sr creatinine < 25%) or a decrease in proteinuria >50% from baseline, and stable serum creatinine (change in serum creatinine < 25%).

#### Steroid resistant nephrotic syndrome (SRNS)

Persistence of nephrotic syndrome despite oral prednisone (1 mg/kg/d) for 16 weeks.

### Treatments

Patients were on follow up for a variable period ranging from 6 months to 5 years. GFR was calculated using a modification of diet in renal disease (MDRD) formula. Patients with nephrotic or nephroto-nephritic syndrome were treated with oral prednisolone (1 mg/kg) for a period of 16 weeks in adults, and 4 weeks in children with subsequent tapering (0.1 mg/kg/week). Patients with crescents were treated with pulse methylprednisolone (15mg/kg/day for 3 days), followed by oral prednisolone at 1 mg/kg for 2 months followed by tapering (0.1mg/kg/week) along with pulse cyclophosphamide (7.5 mg/kg/pulse) for 6 week. A patient with no recovery from dialysis at third months, fourth pulse was omitted.

### Statistical methods

The statistical analysis was carried out using Statistical package for social sciences (SPSS Inc., Chicago, IL, version 15.0 for Windows). All quantitative variables were assessed using measures of central location and measures of dispersion. For normally distributed data, means were compared using student's t-test for two groups. One-way analysis of variance (ANOVA) was used for more than two groups. Qualitative or categorical variable were described as frequencies and proportions. Proportions were compared using the chi-square test wherever applicable. All statistical tests were two-sided and performed at a significance level of α = 0.05. This study was approved by Institutional Review Board (IRB)/Ethics committee-PGIMER Chandigarh (Letter No. Histopathology/13/2088).

### Consent

Written informed consent was obtained from the patient for the publication of this report and any accompanying images and blood/plasma/serum sample was also collected for further serological/genetic investigations with informed consent.

## Results

C3GP comprised 0.7% (n-31) of total native renal biopsies in five years (4565). There were 13 cases (0.3%) of DDD and 14 cases of C3GN (0.3%) of total native kidney biopsies. Four cases (0.1%) had overlapping features between DDD and C3GN and could not further classified.

Patients of C3GP were mostly male in cases diagnosed as C3GN (M: F = ~4:1) and DDD (M: F ratio - 2:1). Within C3GP, most of the patients with DDD had nephrotic syndrome at presentation whereas most of the cases of C3GN had nephroto-nephritic presentation. Further, hypertension and oliguria at presentation was more often seen in C3GN when compared to DDD. Dyslipidemia was seen only in DDD. Four patients of DDD and two patients of C3GN had advanced renal failure at presentation. However, these differences were not statistically significant Table 1.

**Table 1:** **Clinical details of patients of DDD and C3GN**

		**DDD n(%)**	**C3GN n(%)**
**Age**	**Mean ± SD(years)**	25.08(±13.09)	33(±16.91)
	**Range(years)**	12-51	14-64
**Sex**	**Male**	8	11
	**Female**	5	3
**Nephrotic**		7/13(53.9%)	4/14 (28.6%)
**Nephrotic/nephritic**		02/13 (15.4%)	08 (57.1%)
**ESRD**		04/13(30.8%)	02(14.3%)
**Hypertension**		2(16.7)	9(64.23)
**Oliguria**		1(8.3)	3(66.7)
**Dyslipidemia**		7	0
**ANA**		0	0
**ANCA**		0	0
**HBsAg**		1(7.69)	0
**HCV**		0	0
**HIV**		0	0
**Low C3 levels**		6(85.71)	0
**Low C4 levels**		1(14.29)	0

### Glomerular pathology of C3GP

#### Dense deposit disease

**Figure 1:**
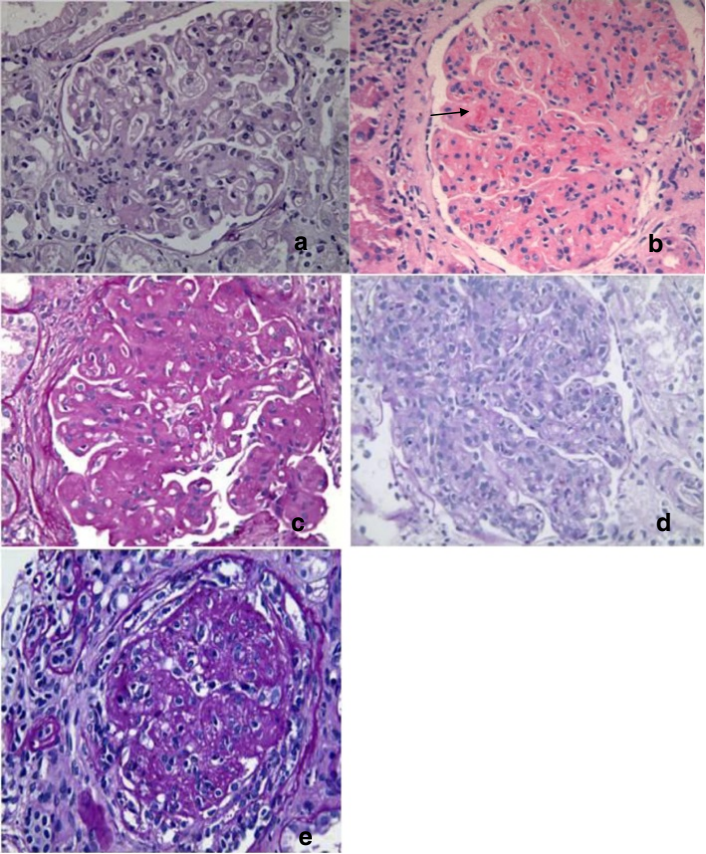
**Photomicrograph shows histomorphological patterns of DDD. (a)** Mild mesangial proliferation. **(b)** Membranoproliferative glomerulonephritis with mesangial refractile material (Arrow). **(c)** Mesangial expansive pattern with mesangial nodules and basement membrane duplication. **(d)** Diffuse proliferative lesion with florid exudation. **(e)** mesangiocapillary pattern with crescent. (a,c,d,e Periodic acid-Schiff and b H&E X40 original).

**Figure 2:**
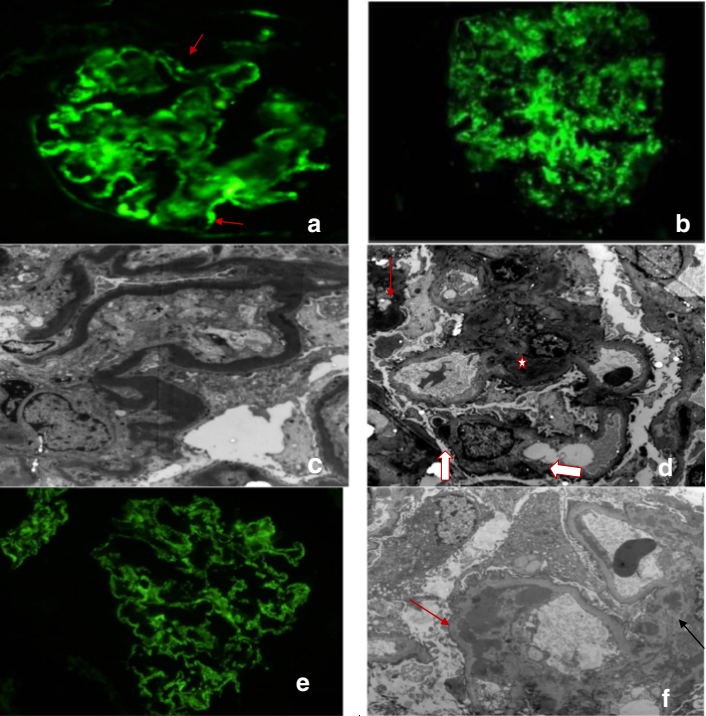
**Photomicrographs showing immunofluorescence pattern and EM of DDD and C3GN. (a)** DDD-IF with C3, coarse staining of glomerular capillary loops and Bowmans capsule. (arrows) **(b)** Predominant mesangial rings with focal glomerular capillaries. **(c)** DDD - EM is showing glomerular capillary intramembranous dense osmiophilic material of variable thickness. **(d)** Osmiophilic material in the mesangium (star), subendothelial (red arrow), subepithelial humps (broad white arrows) (Uranyl acetate, b-2800, c-X36,00). **(e)** C3GN - IF Intense granular positivity in the mesangium and glomerular membranes. **(f)** EM - Subendothelial (red arrow) and mesangial (black arrow) (Uranyl acetate,X10,000).

In cases of DDD, classical MPGN pattern was the predominant pattern seen in 7 cases (58.33%). DPGN like or exudative picture was seen in 2 cases (15.38%), non nodular mesangial expansive in another 2 and nodular mesangial expansive pattern in 1 case. Four cases (30.77%) had crescents in <50% of the total number of glomeruli, but none showed crescentic glomerulonephritis. Refractile membranes and material in mesangium noted in 7 cases (58.3%). Glomerular endothelial swelling seen in 30.77% of cases. The MPGN pattern was the predominant in both paediatric and adult groups. Paediatric patients had more often DPGN like/exudative lesions and frequently had crescents when compared to adults cases of DDD. Focal collapse noted in two cases Figures 1 and 2, Tables 2 and 3.

**Table 2:** **Histomorphological patterns in glomeruli of DDD and C3GN**

**Sr. No**	**Dense deposit disease**				**C3GN**
	**Pattern name**	**Number of cases n(%)**	**Paediatric group n(%)**	**Adult group n(%)**	**Number of cases n(%)**
1	Membranoproliferative	7 (58.33%)	2 (50%)	5 (62.5%)	5 (35.71%)
2	Mesangial expansive*	3 (25%)	1 (25%)	2 (25%)	5** (35.71%)
3	DPGN-like or exudative	2 (16.67%)	1 (25%)	1 (12.5%)	3 (21.43%)
4	Crescentic	0	0	0	1 (7.14%)

Note; *Includes nodular and non nodular mesangial expansive patterns.

**Includes 3 (21.43%) cases of non nodular and 2 (14.29%) cases of nodular mesangial expansive patterns.

**Table 3:** **Clinical follow-up of patients of DDD and C3GN**

	**DDD n (%) TOTAL 13 cases**	**C3GN n (%) TOTAL 14 cases**
**Nephrotic syndrome**	7/13 (53.9%)	4/14 (28.6%)
	No CR/PR	1 (9.05%) achieved CR
	7 (100%)-SRNS	2 (18.1%) achieved PR
		1 (25%) SRNS
**Nephrotic-nephritic presentation**	2/13 (15.4%)	8 (57.1%)
	No CR/PR	No CR/PR
	No Progression to ESRD	No Progression to ESRD
**Advanced renal failure**	4/13 (30.8%)	2 (14.3%)
	No CR/PR	1 (50%)-Complete remission
	4 (100%)-Progression to ESRD	1(50%)-Progression to ESRD

Note; CR-Complete remission; PR-Partial remission; ESRD-End stage renal disease.

EM examination showed osmiophilic continuous or segmental intramembranous and mesangial electron densities in all cases. They were linear, rounded or sausages shaped and were of variable lengths. Some capillary loops were completely involved. Subepithelial humps of similar osmiophilic material observed in 4 cases. One case showed markedly thinned out basement membrane. In addition, material with lesser electron densities as seen in C3GN was seen in two cases with associated fibrillosis of matrix. There were endothelial swelling in 4 cases, and basement membrane remodelling observed in 7 cases. Foot process effacement of podocytes and actin condensation seen in all cases Figure 2.

#### C3 glomerulonephritis

In cases of C3GN, both mesangial expansive and MPGN patterns were commonly seen in 5 cases each (35.71%). Mesangial expansive pattern included non nodular mesangial expansive pattern (n-3, 21.42%) as well as nodular mesangial expansive pattern (n-2, 14.29%) followed by DPGN-like or exudative (n-3, 21.43%). One case (7.1%) had crescentic glomerulonephritis whereas 4 cases (28.57%) had crescents though amounting to < 50% of the total number of glomeruli. Refractile membranes and material in mesangium noted in 7 cases (50%). Glomerular endothelial swelling seen in 2 cases (14.29%) Table 2.

**Figure 3:**
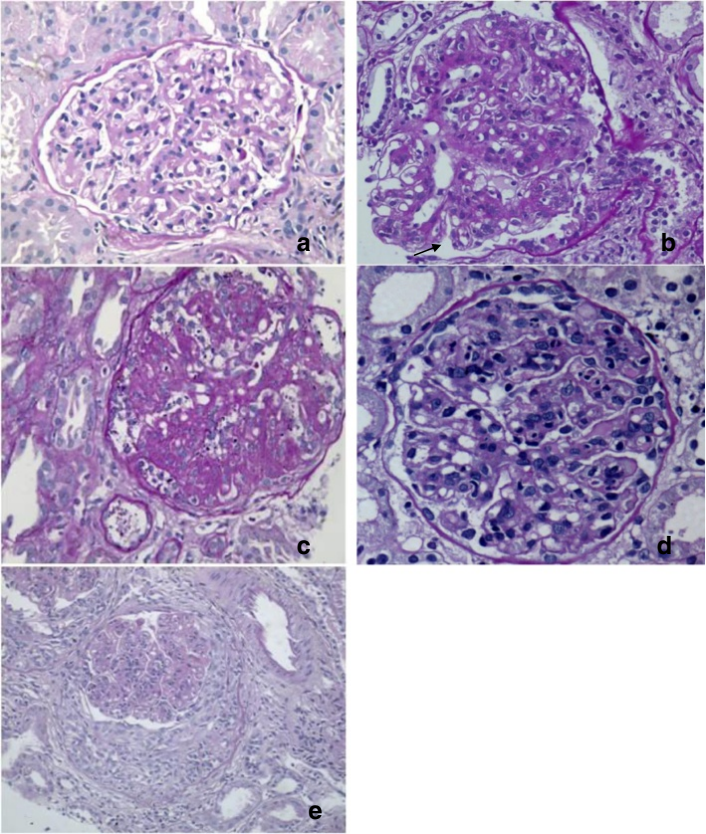
**Photomicrograph shows Histomorphological patterns of C3GN. (a)** Mild mesangial proliferation. **(b)** Membranoproliferative pattern with basement membrane splitting (Arrow). **(c)** Mesangial expansive pattern with mesangial nodules. **(d)** Diffuse proliferative lesion with florid exudation **(e)** Mesangiocapillary pattern with crescent (Periodic acid Schiff H&E, a-d, X40 original, e-X20 original).

Comparing the histomorphological patterns within C3GP, mesangial expansive and MPGN patterns were more often seen in C3GN whereas MPGN with endocapillary proliferation was more often seen in DDD. Crescentic glomerulonephritis was seen only in C3GN, but the proportion of cases showing crescents was equal in both. The percentage of cases showing DPGN like or exudative pattern was nearly equal in these two groups. Retractile membranes and refractile material in the mesangium were seen in about half of the cases of each of these two categories Figures 2 and 3, Table 2.

EM examination of cases showed dominant mesangial deposits of immune complex type in all cases along with subendothelial deposits in 12 cases. Subepithelial immune complex types of humps were seen in 1 case only. Endothelial swelling was observed in 3 cases and basement membrane remodelling in 4 cases. Osmiophilic electron densities like that seen in DDD was seen focally in two cases. Foot process effacement of podocytes and actin condensation was seen in all cases.

#### Glomerular sclerosis

Variable number of sclerosed glomeruli were present in 58.33% of cases of DDD (Zero-7, <10%-2, >50%-3, >90%- 1) and 57.14% among C3GN(Zero-6, <10%-4, >50%-3, >90%- 1). However, it was not statistically significant.

#### Tubulointerstitial changes

Within C3GP, tubulo-interstitial changes were more frequent and more advanced in DDD compared to C3GN. None of the cases of DDD had normal tubulo-interstitium whereas about a quarter of cases of C3GN did not show any tubulo-interstitial changes. The presence of interstitial foam cells in DDD was more than twice compared to that seen in C3GN.

#### Follow-up

Four (28.6%) DDD and two (15.4%) C3GN cases had advanced renal failure at presentation. The outcome of patients with DDD and C3GN is mentioned in Table 3.

None of the patients of DDD showed partial or complete remission and four of them progressed to ESRD. Renal biopsies of patients who progressed to ESRD had more than 50% sclerosed glomeruli. C3GN patients with nephrotic presentation showed complete remission in one and partial remission in two. One C3GN patient who had advanced renal failure and crescentic morphology also showed complete remission (Table 3).

## Discussion

C3GP comprise 0.7% all native renal biopsies received during five-year study period; DDD and C3GN were equally represented ( 0.3% each) and 0.1% could not be further categorized because significant overlap on electron microscopy. Majority of DDD patients were younger as compared to patients with C3GN. Among DDD, 69.23% of patients were <25 years of age and 30.77% were in paediatric age group. The mean age at diagnosis was 25.08 years. Prevalence of DDD among patients in the younger group is comparable to studies by Lu et al. [17] and Smith et al. [11] who studied mostly children and young adults. However, Nasr et al. had only 43.8% in paediatric or adolescent age group. In C3GN, the mean age at diagnosis was 33 years with age range of 14-64 years [12]. Five patients (35.71%) were less than 25 years of age. In the study of C3GN by Sethi et al. [8], age ranged from 8 to 73 years (mean, 42.5 years). Median age at onset was 29.9 years (range 7–70) [18] which is comparable to the present study (Table 1).

There are difference in renal parameters at presentation in present cohort and ones from west reported in literature. In DDD, majority (53.9%) of the patients had nephrotic syndrome, 15.4% had nephroto-nephritic and 30.8% had advance renal failure at presentation. Mean serum creatinine was 4.65 mg/dl and hypertension was seen in 16.7%. In a study by Habib et al. [19], all patients of DDD had proteinuria, and hypertension was seen in 27% as was observed in the present study. However, in a study by Nasr et al. nephrotic syndrome was present in 33.3% of patients that is significantly less than patients with nephrotic syndrome in present study. In their study, mean serum creatinine was 2.2 mg/dl and renal failure was seen in 59.4% of patients. In a study by Smith et al. [11] patients of DDD had proteinuria in 90%, nephritic syndrome in 34% and hypertension in over 50%. In comparison with these two studies, patients of DDD in the present study, more often presented with nephrotic syndrome with proteinuria >3g and higher serum creatinine values.

Similarly, patients with C3GN in had more often advanced renal failure and nephrotic range proteinuria as compared to other studies. In C3GN, nephrotic syndrome was seen in 28.6%, nephroto-nephritic in 57.1% and advanced renal failure in 14.3%. Proteinuria(24hr) ranged from 1.2g to 7.2g/24 h (mean 3.98g/24 h). In a study by Sethi et al. [8] all patients had hematuria and proteinuria. 24hr urinary protein ranged from 0.615g to 15g/24h (mean 5.76g/24h). In their study, serum creatinine at presentation ranged from 0.6 to 3.1 mg/dl (mean 1.5 mg/dl) that was lower than observations in the present study (mean 4.19 mg/dl). Hypertension was seen in 64.23% in the present study whereas nine out of their twelve patients (75%) were hypertensive. In a study by Servais et al. [18] mean proteinuria was 3.3 g/day (0.2–9.0); nephrotic syndrome was seen in 68.42% of cases and nephritic syndrome in 63.15%. Hypertension was seen in 47.37%.

Histomorphologic spectrum of C3GN has expanded over the years. In present study, the most common pattern in biopsies with DDD was MPGN accounting for over a half of the cases. Next common was mesangial expansive pattern accounting for one-quarter of cases. The least common pattern is that of exudative or DPGN like pattern (16.67%). Though crescents were seen in about one-third of cases (30.77%), none had crescentic glomerulonephritis. In a study by Walker et al. mesangial expansive pattern was the predominant pattern seen in 45% of cases followed by MPGN pattern. Crescentic glomerulonephritis was seen in one-fifth and. DPGN like pattern in 12% of their cases. However, distribution of various morphological subtypes in adults was similar to that observed by Nasr et al. [12] in their adult patients Tables 4 and 5.

**Table 4:** **Comparison of morphological patterns of DDD and C3GP**

		**DDD**			**C3GN**			
**Sr.No.**	**Pattern name**	**Present study**	**Walker et al.** **[** 20 **]**	**Nasr et al.** **[** 12 **]**	**Present study**	**Sethi et al.** **[** 7 **]**	**Fakhouri et al.** **[** 5 **]**	**Servais et al.** **[** 8 **]**
1	**Membranoproliferative**	58.33%	25%	55.6%	35.71%	80%	75%	68.42%
2	**Mesangial proliferative**	25%	45%	27.8%	35.71%	-	25%	31.58%
3	**DPGN-like or exudative**	16.67%	12%	16.7%	21.43%	20%	-	-
4	**Crescentic**	0	18%	0	7.14%	-	-	-

**Table 5:** **Comparison of morphological patterns of DDD in paediatric and adult group**

**Sr. No**	**Pattern name**	**Paediatric group**		**Adult group**		
		**Present study (%)**	**Nasr et al.** **[** 12 **]**	**Present study**	**Nasr et al.** **[** 12 **]**	**Walker et al.** **[** 20 **]**
			**(%)**	**(%)**	**(%)**	**(%)**
1	Membranoproliferative	50	28.6	55.56	55.6	25
2	Mesangial proliferative	25	28.6	22.22	27.8	45
3	DPGN-like or exudative	25	21.4	11.11	16.7	12
4	Crescentic	0	21.4	0	0	18

However, when paediatric patients of DDD are compared to paediatric patients of the series by Nasr et al. [12] more number of patients in the present series had MPGN pattern and lack crescentic DDD pattern. Fifty percent of paediatric patients in the present study had few crescents (less than 50%) These differences could be due to ethnic differences or small sample size of paediatric patients Tables 4 and 5.

In the present study, MPGN pattern was present in lesser number of cases of C3GN when compared to the series by *Fakhouri et al.* [6], *Sethi et al.* [8], and *Servais et al.* [9]. DPGN like pattern was equally common (20%) in study material when compared to Sethi et al. Mesangial proliferation pattern, crescentic GN and cases with lesser number of crescents was much more often seen in present study when compared with *Fakhouri et al.* [6], *Sethi et al.* [8], and *Servais et al.* [9] which do not observe any crescents (crescentic/-few crescents), DPGN pattern at all Tables 4 and 5.

## Conclusion

In conclusion, incidence of C3GP is 0.7% of all native biopsies with almost equal representation of DDD and C3GN. Most of the cases of DDD have nephrotic presentation whereas C3GN have nephroto-nephritic presentation; advanced renal failure at presentation is often seen in DDD. Morphological spectrum revealed proliferative lesions like MPGN and MCGN patterns are equally common in C3GN whereas MPGN was the dominant pattern in DDD. Limited follow-up revealed response to therapy only in C3GN (one-third cases). There was a progression to ESRD in one-third cases of DDD and only 10% cases of C3GN. However, longer follow-up is needed to understand the progression of the subcategories of C3GP. Autoimmune/genetic work needs to do for the diagnosis refinement of variants or cases with ambiguous findings.

## Abbreviations

C3GP-C3: Glomerulopathy
DDD: Dense deposit disease
C3GN: C3 glomerulonephritis
MPGN: Membranoproliferative glomerulonephritis
ESRD: End-stage renal disease
